# Power Up Plant Genetic Research with Genomic Data

**DOI:** 10.3390/ijms24086876

**Published:** 2023-04-07

**Authors:** Man-Wah Li, Sachiko Isobe, Hon-Ming Lam

**Affiliations:** 1 Centre for Soybean Research of the State Key Laboratory of Agrobiotechnology and School of Life Sciences, The Chinese University of Hong Kong, Hong Kong, China; manwah.li@yale.edu; 2 Department of Molecular, Cellular and Developmental Biology, Yale University, New Haven, CT 06510, USA; 3 Laboratory of Plant Genetics and Genomics, Kazusa DNA Research Institute, Kisarazu 292-0828, Chiba, Japan; sisobe@kazusa.or.jp

The official debut of the reference genome of *Arabidopsis thaliana* in 2000 [1] marked the genesis of the plant genome era. Yet, limited by the sequencing technology and the complexity of most plant genomes, reference-grade genomes have only been available for a few important plant species in the two decades since [2]. In the past few years, the rise of third-generation sequencing (or so-called long-read sequencing) platforms has accelerated the assembly of genomes at the chromosome level [2,3,4]. Up until now, it has been estimated that, out of the ~40,000 green plant species, less than 0.2% have had their genomes assembled at the chromosome level [2,3]. Despite this relatively small achievement, these published genomes have already been able to advance genome-guided research, including, but not limited to, population genomics, transcriptomics, proteomics, meta-genomics, meta-transcriptomics and epigenomics. In this Special Issue, we have collected eight such plant genomics studies from dedicated researchers.

The plant genome size ranges from several tens of megabases to hundreds of gigabases [5]. Nevertheless, the number of protein-coding genes is not strictly correlated with the genome size, as most plant genomes are predicted to have similar numbers of protein-coding genes, averaging around 30,000 [5]. For uncharacterized genomes, an easy way to identify functional genes is to search for known gene families based on sequence homology and previous research of model organisms. The sucrose nonfermenting 2 (Snf2) family of proteins have previously been identified to modulate reproductive organ development in the model plant Arabidopsis, making them potential candidates for affecting crop yield. Adopting the hidden Markov model (HMM)-based approach, Chen et al. identified 38 high-confidence Snf2 protein-encoding genes in the genome of barley (*Hordeum vulgare*) [6]. These *Snf2* genes can be classified into 6 groups and 18 subfamilies phylogenetically. Some of these *Snf2* genes were found to be differentially expressed in floral organs, supporting the notion that they are important for determining the yield of barley. Another study by Lin et al. successfully identified, in the pearl millet genome, 208 putative MYB-coding genes [7], a transcription factor family participating in diverse biological functions. Substitution rate analyses of orthologous *MYB* pairs suggested that they were under purifying selection during domestication. Expression analyses of 11 selected *MYB* genes revealed that their expressions were responsive to stress treatments, resembling the characterized *MYB* genes from other organisms. This catalog of *MYB* genes would be useful for supporting further research of this gene family in pearl millet.

Whole-genome resequencing, in theory, could detect any small genomic variations in the genome at low cost. Thus, it is a powerful tool for genotyping genetic populations for forward genetics studies. In most cases, genetic markers are no longer the limiting factor for genetic mapping; instead, the number of recombination events within the population is the limiting factor. Guan et al. resequenced 200 F2 eggplants from an interspecific cross (*Solanum melongena* × *S. incanum*) for the construction of a recombinant bin map [8]. Using the phenotypic data of 172 F2:3 lines and this recombinant bin map, a major quantitative trait locus (QTL) was identified corresponding to the leaf vein color and fruit pericarp color. Using the single-nucleotide polymorphism (SNP) and insertion/deletion (INDEL) information from F2 resequencing, the same genomic region was also rediscovered. Similarly, Liang et al. made use of 267 maize double-haploid (DH) lines and 336 inbred lines to map the genomic loci associated with the ear shank length, a trait determining the grain yield and kernel dehydration rate [9]. Using previously available bin maps and SNP data, they successfully identified 16 QTLs and 23 SNPs associated with the ear shank length via QTL mapping and genome-wide association studies (GWAS), respectively. By overlapping the two sets of data, this group identified four loci common to all the study populations for further investigation. Through the analysis of transcriptional dynamics during maize shank elongation, the researchers narrowed down the candidate genes to a final list of 12 differentially expressed genes.

Transcriptome analyses have been adopted widely to study plant–pathogen interactions. By integrating histology, transcriptomics and metabolomics, Zhou et al. tapped into the changes in walnut (*Juglans regia* L.) upon infection by *Phomopsis capsica* [10]. They discovered that *P. capsica* can lead to severe destruction of the vasculature of the infected walnut branches. The association of differentially expressed genes with differentially expressed metabolites demonstrated that *P. capsica* infection disturbs the metabolism of carbohydrates, amino acids and some secondary metabolites.

Apart from simply examining the host response toward the pathogen, variants of transcriptomic analyses have been used to delineate bidirectional interactions. For example, the meta-transcriptome can be used to identify the pathogen’s own pathogen. Fungal diseases are the major threats to rice harvest. Biological control of fungal diseases using mycoviruses can be a sustainable solution. To identify potential mycoviruses as suitable biological control agents for fungal diseases in rice, He et al. [11] sequenced the meta-transcriptome of 343 strains of fungal pathogens of rice that are potentially affected by viruses. In total, 68 mycoviruses were identified from 682 contigs, with 42 of them never described before. Associated with the stunted growth of the fungal pathogens, the mycoviruses identified in this study could potentially be new biological control agents. Another study made use of dual transcriptome analyses to study the interactions between *Colletotrichum higginsianum* and *Arabidopsis thaliana*, the former being a fungal pathogen of the latter, which is the model for cruciferous plants [12]. Autophagy has been demonstrated to be important for fungal pathogenicity. The host, *Arabidopsis*, was inoculated with either wildtype *C. higginsianum* (ChWT) or a mutant with impaired autophagy (*Chatg8Δ*). Through dual transcriptome analyses, it was shown that *Chatg8Δ* significantly affected the melanin pathway, in which a deletion in the most enriched gene encoding trihhydroxynaphthalene reductase (*THR1*) could lead to the loss of pathogenicity.

A well-annotated reference genome is always an important foundation for other omics studies. Rehman et al. analyzed the salt-induced membrane proteomic changes in the roots and leaves of a salt-sensitive soybean cultivar using a label-free quantitation strategy, and identified 127 differentially expressed membrane proteins [13]. These proteins are involved in ion transport, protein transport, ATP hydrolysis, protein folding, receptor kinases, etc. Verified with previously published RNA-seq data, this study demonstrated the importance of solute transport and sensing in salt stress responses.

This Special Issue explored some new areas in omics research in plants, demonstrating the power of omics strategies and their potential applications in improving crop production. These studies collectively provide a large amount of valuable omics data and information for further investigation.
